# The effect of *Zataria multiflora* and carvacrol on wheezing, FEV1 and plasma levels of nitrite in asthmatic patients 

**Published:** 2017

**Authors:** Azam Alavinezhad, Mahdiyeh Hedayati, Mohammad Hossein Boskabady

**Affiliations:** *Neurogenic Inflammation Research Center, Department of Physiology, School of Medicine, Mashhad University of Medical Sciences, Mashhad, Iran*

**Keywords:** Asthma, Zataria multiflora, Carvacrol, Forced expiratory volume in one second, Wheezing, Nitrite

## Abstract

**Objective:**

The preventive effect of* Zataria multiflora* in animal models of asthma has been reported. In the present study, its effect on wheezing, forced expiratory volume in 1 second, and plasma nitrite (NO^2-^) in asthmatic patients was studied.

**Materials and Methods:**

In this study, forty asthmatic patients including 15 males and 25 females (aged 45.8±6.21 years) were randomly allocated in four groups including placebo group (P), and three treatment groups which received two doses of *Z. multiflora* (groups Z low and Z high that received 5 and 10 mg/kg/day, respectively) and carvacrol (group C treated with 1.2 mg/kg/day). All patients were treated for two months in a double-blind manner. At three time points (before starting the treatment (pretreatment), and one and two month after treatment), wheezing during day and exercise, forced expiratory volume in 1 second and NO^2-^ were measured.

**Results:**

Day wheeze and exercise wheeze were significantly reduced in treated groups with carvacrol and both doses of *Z. multiflora* compared to pretreatment (p<0.05 to p<0.01). However, FEV_1_% was significantly increased in treated groups with carvacrol and both doses of *Z. multiflora* (p<0.05 to p<0.001). Plasma level of NO^2-^ was also significantly decreased in Z high and carvacrol groups after two months of treatment (p<0.01 to p<0.001). However, most of evaluated parameters in placebo group did not show significant changes during the study.

**Conclusion:**

*Z. multiflora* and its constituent, carvacrol, improved FEV_1_% while reduced wheezing and plasma level of NO^2-^ in asthmatic patients. Therefore, a possible therapeutic potential for this plant and its constituent, carvacrol could be suggested to be used against asthma.

## Introduction

*Zataria multiflora* (*Z. multiflora*) essential oil has phenolic (such as thymol, carvacrol and linalool) and non-phenolic (such as p-cymene, γ-terpinene and α-pinene) constituents, as well as flavonoids, tannins, resins and saponins. The percentage of these constituents alters in different regions. Carvacrol (2-methyl-5-(1-methylethyl)-phenol) is the main constituent of the essential oil of this plant (Saleem et al., 2004 ▶) which no side effects have been reported so far. This compound was approved by the FDA for use in food industry (De Vincenzi et al., 2004 ▶). 

In traditional medicine, *Z. multiflora* is used for treating respiratory diseases. This plant has many therapeutic properties such as antiseptic (antimicrobial and anti-fungal) and antitussive (Aynehchi 1986 ▶; Hosseini et al., 2016 ▶; Jafari et al., 2003 ▶; Mansoori et al., 2002 ▶; Mansour et al., 2010 ▶; Saei-Dehkordi et al., 2010 ▶), oxidative stress modulatory activities (Babaie et al., 2007 ▶; Jaffary et al., 2000 ▶; Nakhai et al., 2007 ▶). This plant has shown stimulatory effect on β2-adrenoceptors, and inhibitory effect on histamine (H1) and muscarinic receptors in guinea pig tracheal chains (Boskabady et al., 2009 ▶; Boskabady et al., 2012a ▶; Boskabady et al., 2010 ▶; Boskabady et al., 2012b ▶; Jafari et al., 2011 ▶). It could also ameliorate bronchial inflammation (Boskabady and Gholami Mhtaj 2014 ▶; Boskabady et al., 2014a ▶; Boskabady and Mahtaj 2015 ▶; Boskabady et al., 2014b ▶) and elevation of Th1/Th2 ratio in sensitized animals (Boskabady et al., 2013 ▶). In lipopolysaccharide-stimulated macrophages, *Z. multiflora* suppressed oxidative stress likely due to its radical scavenging activity and through reduction of NO production (Kavoosi 2011 ▶).

Several pharmacological properties were reported for carvacrol in previous studies such as antioxidant (Chen et al., 2009 ▶), anti-inflammatory, antimicrobial (Nostro and Papalia 2012 ▶) and antitumor effects (Jaafari et al., 2012 ▶). Carvacrol can reduce inflammation by inhibition of cyclooxygenase-2 and consequently decreasing prostaglandin E2 as well as modulating TNF-α, IL-6, iNOS and IL-10 levels (Aristatile et al., 2013 ▶; Kara et al., 2015 ▶; Landa et al., 2009 ▶; Lima et al., 2013 ▶). In sensitized guinea-pigs treated with carvacrol, the serum levels of IL-4, endothelin, IgE and eosinophil peroxidase decreased and IFN-γ increased (Boskabady et al. 2014b ▶; Jalali et al., 2013 ▶). In addition, carvacrol decreased total WBC number, eosinophil, serum levels of IL-8 and malondialdehyde in an animal model of COPD (Mahtaj et al., 2015 ▶). An *in vivo* study on human monocytes showed that *Z. multiflora* essential oil, carvacrol and thymol can reduce NOS and NOX activities as well as production of NO and H_2_O_2_ (Kavoosi et al., 2012 ▶). Carvacrol also showed inhibitory effects on histamine and muscarinic receptors (Boskabady et al., 2011 ▶; Boskabady et al. 2012b ▶) and demonstrated a potent relaxant effect on tracheal smooth muscles (Boskabady and Jandaghi 2003b ▶). 

Asthma is a chronic inflammation of airways which leads to respiratory symptoms such as wheezing, coughing, shortness of breath and chest tightness ( Bateman ED et al., 2008 ▶). Pulmonary function tests (PFT) are usually less than 80% predicted values in asthma (Bousquet 2000 ▶). Inflammatory markers such as the level of NO^2-^ can be related to the degree of airway inflammation and are involved in the pathogenesis of asthma (Ekmekci et al., 2004 ▶). One of the oxidative products of NO is NO^2-^ which significantly increases in asthma and chronic obstructive pulmonary diseases (Kanazawa et al., 1998 ▶). According to Global Initiative for Asthma (GINA), 300 million persons are affected by asthma worldwide and this number will reach 400 million until 2025 (Bousquet et al., 2007 ▶). 

Asthma has no certain cure and needs regular and continuous management. Thus, development of more effective treatments for asthma is required. 

With regard to the preventive effect of *Z. moltiflora* and carvacrol shown in animal models of asthma, the goal of this study was to examine the preventive effect of treatment with the plant and its constituent on wheezing, FEV_1_ and lung inflammation marker (NO^2-^) in asthmatic patients during a two-month treatment period.

## Materials and Methods

**Subject selection**

According to GINA guideline, forty moderate to severe asthmatic patients were invited from asthma clinic, Mashhad University of Medical Sciences, Mashhad, Iran. 

Inclusion criteria for the patients were: diagnosis with asthma by the physician, having two or more of symptoms such as cough or chest tightness at rest, nocturnal or early morning wheeze and wheeze or cough during exercise, FEV_1_ and PEF (Peak expiratory flow) less than 60% predicted values. Exclusion criteria were: having a history of other respiratory diseases, presence of respiratory infections, having cardiovascular diseases or diabetes, pregnancy and being younger than 20 or older than 70 years old. During the treatment period, patients used their previously prescribed drugs (i.e. corticosteroid inhalers, salbutamol and oral theophylline). This clinical trial was registered in Iranian Registry of Clinical Trials (IRCT Code: IRCT 2014101519546N1) and it was also approved by the Ethics Committee of Mashhad University of Medical Sciences (Ethics approval code: 910681). In addition, all subjects signed a written informed consent.

**Treatment groups**

The patients were allocated to four groups (n=10) by block randomization as block size was 8 and blocks were selected by random numbers Table. Groups were as follow:

1. Placebo group (P)
2. Z low group which received 5 mg/kg/day *Z. multiflora*
3. Z high group which received 10 mg/kg/day *Z. multiflora*
4. C group which received 1.2 mg/kg/day carvacrol.

The doses of the extract and carvacrol were chosen according to the previous studies done on these agents in animal models of lung diseases (one tenth of animal doses were chosen for human subjects), (Gholami Mahtaj et al., 2015 ▶). Patients in each treatment group consumed prepared pharmaceutics 3 times a day for two months along with their routine medications. The manner of administration was double blind. Evaluation of wheezing, FEV_1_ and blood samples were performed at three time points namely, pre-treatment (Time O), one month after the treatment (Time I) and two months after the treatment (Time II).

**Pharmaceutical preparations**

The extract of *Z. multiflora* was purchased from Gieah-Essans Co. (Gorgan-Iran) which contained 20% alcohol, 55.4 mg/100 ml thymol, 7.7 mg/100 ml carvacrol, and 63.2 mg/100 ml total phenol. For preparation of the elixirs for Z low and Z high groups, 116 mg/5 ml and 232 mg/5 ml of the extract were dissolved in simple syrup, respectively. The amount of carvacrol in the elixir was measured by GC method (Liolios et al., 2009 ▶). The GC analysis was performed using a Varian CP-3800 equipped with FID detector, fused-silica column (CP-Sil 8CB, 50 m×0.25 mm, film thickness 0.12 μm). For preparation of the placebo elixir, 5% alcohol was dissolved in simple syrup (80% w/v sucrose). 

Carvacrol pharmaceutical grade (90%) was purchased from Ji’An HaiRui Natural Plant Co. (China). Pellets were produced by coating carvacrol onto the nonpareil beads (850–1180 μm) using fluidized bed coater (Wurster insert, Werner Glatt, Germany). For preparation of 80% (w/v) of carvacrol, 5% hydroxypropylmethyl cellulose (HPMC) and 2% Talc were dispersed in absolute ethanol. The suspension was sprayed onto nonpareils using fluidized bed coater. The suspension was stirred throughout the layering process. The carvacrol layering process was carried out to produce pellets with about 7.5 and 11.75% (w/w) carvacrol load. After coating, the pellets were re-coated with HPMC 5% solution and fluidized for about 5 min and then were kept in an oven for 2 hr at 40°C. The amount of carvacrol in pellets was measured by GC (with above-mentioned properties). 

Although all drugs were used during 3 months, an accelerated stability study (40°C ± 2°C/ 75% ± 5% RH) was done for a period of 6 months (Bajaj et al., 2012 ▶). The results displayed no significant changes throughout this period. 

**Assessment of wheezing and FEV**_1_

For assessment of the severity of day wheeze (DW) and exercise wheeze (EW), a questionnaire (in Farsi) was used (Boskabady and Farhadi 2008 ▶; Boskabady et al., 2007 ▶; Boskabady and Kolahdoz 2002 ▶; Masjedi MR et al., 1989 ▶). Measurement of FEV_1_ was performed based on standards outlined by the American Thoracic Society (ATS) using a spirometer with a pneumotachograph sensor (Model ST90, Fukuda, Sangyo Co. Ltd., Japan). Prior to measurement of FEV_1_, the technique was taught by the operator. FEV_1_ was measured for three times in a sitting position while patients were using nose clips. Finally, the best values among three measurements, were selected. 

**Blood sampling and NO** ^2-^ **measurement**

Blood samples were collected and the plasma was separated for NO^2-^ measurement. Measurement of nitrite was done using Griess Reagent System kit (Cat.# G2930).

**Data analysis**

The results were presented as mean ± SD. The data were analyzed by SPSS (version 11.5, SPSS Inc. USA). Significance level was considered at p<0.05. The comparisons within each group during three times of the study were done using a General Linear Model for Repeated Measures, with time as within-subjects. The Bonferroni test was performed for pairwise comparisons. The comparison of wheezing was done by sign test. The comparison of percentage of changes of measured values during two month treatment among treated and placebo groups were carried out by Independent t-test.

The percentage of changes of each variable at time I or II relative to time 0 was calculated using the following equations:

$\frac{\left(\mathrm{Value at time I or II}-\mathrm{Value at time 0}\right)\times \mathrm{100}}{\mathrm{Value at time 0}}$

Calculation of percentage of changes of values at time II relative to time I, was done by the following equation:

$\frac{\left(\mathrm{Value at time I}-\mathrm{Value at time I}\right)\times \mathrm{100}}{\mathrm{Value at time I}}$

## Results

**Demography**

Forty patients were divided into four groups. In placebo group, 10 patients (4 males and 6 females) with an average age of 46.1 ± 9.6 years old, a family history of asthma of 60%, a history of active smoking of 10% and an asthma severity of 4.1 ± 2.37 were included. The patients of Z low group (n=10) were 3 males and 7 females with an average age of 42.5 ± 1.37 years, a family history of asthma of 80%, a history of active smoking of 10% and an asthma severity of 4.5 ± 3.06. Asthmatic patients in Z high group (n=10) were 4 males and 6 females with an average age of 48.2 ± 8.23 years, a family history of asthma of 60%, a history of active smoking of 20% and an asthma severity of 5.3 ± 3.43. In the group treated with carvacrol also 10 patients (4 males and 6 females) with an average age of 46.6 ± 1.23 years, a family history of asthma of 80%, a history of active smoking of 0% and an asthma severity of 4.1± 3.31 participated (Table 1). Asthma severity did not show statistically significant differences among the four groups. 

**Table 1:** Demographic information of the patients in different groups

**Characteristic**		**P**	**Z low**	**Z high**	**C**
**Number of patient**		10	10	10	10
**Sex**	**Male** **Female **	40% 60%	30% 70%	40% 60%	40% 60%
**Age (year)**		46.1±9.6	42.5±1.37	48.2±8.23	46.6±1.23
**Height (cm)**		162±7.86	162.7±8.93	163.9±6.36	159±8.48
**History of smoking**	**Active** **Passive**	10% 30%	10% 20%	20% 20%	0 50%
**Family history of asthma**		60%	80%	60%	80%
**Asthma severity**		4.1±2.37	4.5±3.06	5.3±3.43	4.1±3.31

The results are presented as mean ± SD. Statistical comparisons were performed using independent T-test.

P; Placebo group, Z low group were treated with 5 mg/kg/day of the extract, Z high group were treated with 10 mg/kg/day of the extract, C group were treated with 1.2 mg/kg/day carvacrol.

**The effects of** ***Z. multiflora*** **and carvacrol on wheezing and FEV**_1_

**Figure 1:**
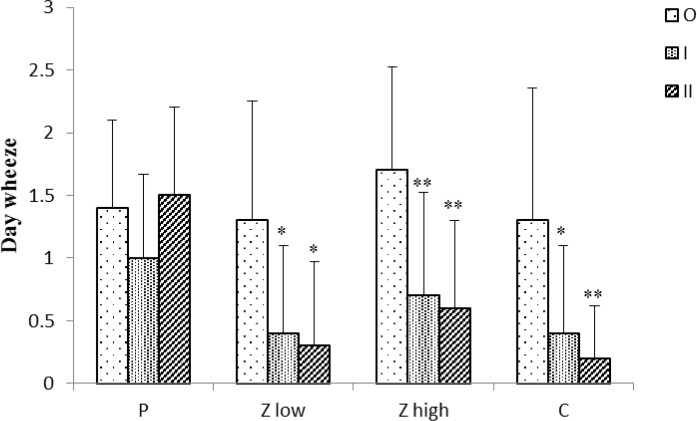
Day wheeze scores for placebo and treated groups (n =10) at three time points (O, I, and II).

**Figure 2:**
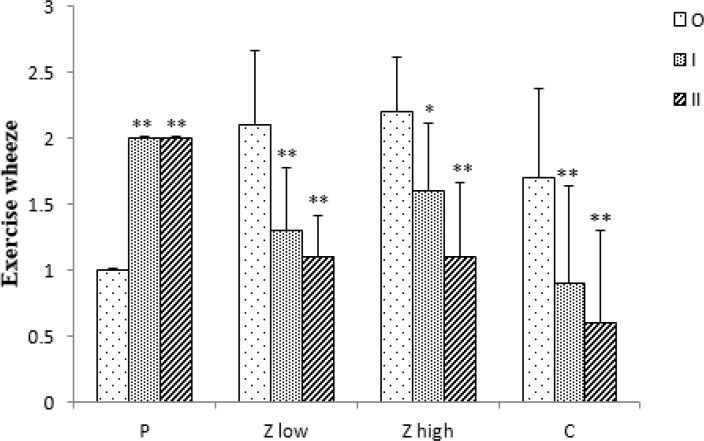
Exercise wheeze scores for placebo and treated groups (n =10) at three time points (O, I, and II).

**Figure 3:**
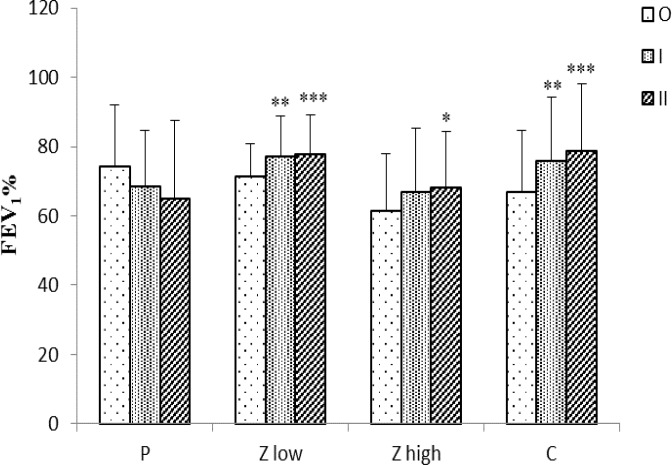
FEV1% for placebo and treated groups (n =10) at three time points (O, I, and II).

In the placebo group, day wheeze (DW) and values of FEV_1_ did not show variations among three time points of the study. However, exercise wheeze (EW) significantly increased at time I and II compared to time O (p<0.01 for both case), (Figure 1-3).

The results are presented as mean ± SD. Statistical comparisons were carried out by sign test.

* p<0.05 and ** p<0.01 show significant differences in data obtained at time I and II from that of time O, respectively.

P: Placebo group; Z low group were treated with 5 mg/kg/day of the extract; Z high group were treated with 10 mg/kg/day of the extract; C group were treated with 1.2 mg/kg/day carvacrol.

Lung wheezing and values of FEV_1_ among treated groups at three time points showed significant changes. Day wheeze in treated groups with carvacrol and two doses of the extract at time I was significantly reduced compared to time O (p<0.05 to p<0.01), (Figure 1a). Day wheeze in all treated groups at time II compared to time O was significantly decreased (p<0.05 to p<0.01), (Figure 1).

Exercise wheeze at both time points (I and II), as compared to time O, was significantly decreased in Z low, Z high and C groups (p<0.05 for Z high at time II and p<0.01 for other cases), (Figure 2).

In addition, values of FEV_1_ significantly increased in Z low and C groups at both time I and II compared to time O (p<0.01 for time I and p<0.001 for time II) and in Z high, only at time II compared to time O (p<0. 05), (Figure 3). 

**The effects of** ***Z. multiflora*** **and carvacrol on the plasma level of NO** ^2-^ **as an oxidative stress and inflammation marker**

**Figure 4:**
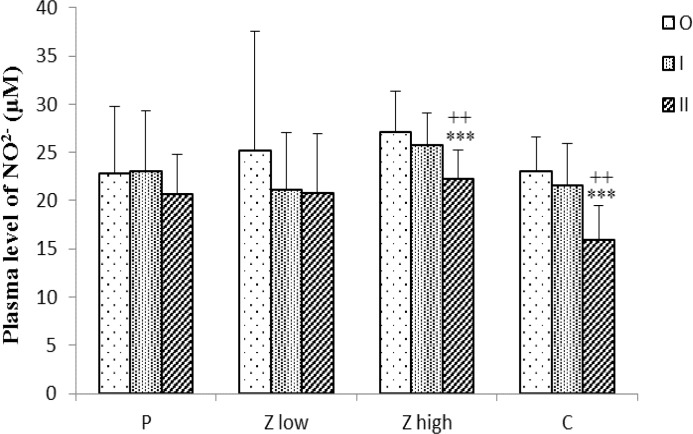
Plasma level of NO^2-^ for placebo and treated groups (n =10) at three time points (O, I, and II).

The plasma level of NO^2-^ in Z high and C groups was significantly reduced at time II compared to time O (p<0.001 for both cases) and at time II compared to time I (p<0.01 for both cases), (Figure 4).

The results are presented as mean ± SD. Statistical comparisons were carried out by sign test.

* p<0.05 and ** p<0.01, show significant differences in data obtained at time I and II from that of time O, respectively.

P: Placebo group; Z low group were treated with 5 mg/kg/day of the extract; Z high group were treated with 10 mg/kg/day of the extract; C group were treated with 1.2 mg/kg/day carvacrol.

The results are presented as mean ± SD. Statistical comparisons were performed using Repeated Measures procedure, with time as within-subjects. Bonferroni test was performed for pairwise comparisons.

* p<0.05, ** p<0.01, and *** p<0.001, show significant differences in data obtained at time I and II from that of time O, respectively.

P: Placebo group; Z low group were treated with 5 mg/kg/day of the extract; Z high group were treated with 10 mg/kg/day of the extract; C group were treated with 1.2 mg/kg/day carvacrol. 

**Comparison of percentages of changes in wheezing, FEV**_1_ **and NO** ^2-^ **during treatment period between treated and placebo groups**

Percent of changes in DW at time I relative to time O for only Z high and at time II relative to time O for all treated groups were significantly higher compared to placebo group (p<0.05 to p<0.01), (Table 2). Percentage of changes in EW in all treated groups (Z low, Z high and C) both at time I relative to time O and at time II relative to time O, were significantly higher than group P (p<0.001 for all cases), (Table 2). Also, Percentage of changes in FEV_1_ in all treated groups (Z low, Z high and C) in both time I and II relative to time O were significantly higher than group P (p<0.05 to p<0.001), (Table 2).

The percentage change of NO^2-^ during 2 months period of treatment only in time II relative to time O for group C which was significantly higher than group P (p<0.05), (Table 2).

**Comparison of percentages of changes in wheezing, FEV**_1_ **and NO** ^2-^ **during treatment period among** ***Z. multiflora*** **and carvacrol-treated groups**

Percentage of changes of EW at time II relative to time O for Z low and Z high groups were significantly lower than group C (p<0.05 and p<0.01, respectively). Percentage of changes in NO^2-^ at time II relative to time I for Z low group was also significantly lower than group C (p<0.01), (Table 2).

**Table 2:** Percentage of changes in day wheeze (DW), exercise wheeze (EW), FEV_1_, and NO^2-^ at time I relative to time O (I/O), time II relative to time O (II/O) and, time II relative to time I (II/I) in treated groups

**Time**	**Group**	**DW**	**EW**	**FEV**_1_	**NO** ^2-^
**I/O**	**P**	-25±35.36 -53.33±50.18 -66.67±37.68* -51.67±47.43	100±0.00 **-**36.67±20.49*** -26.67±23.83*** -53.33±37.52***	-6.05 ±12.51 7.9±7.16 ** 9.84±19.68* 15.17±15.74***	14.05±64.02 **-**4.33±37.79 -4.23±8.91 -6.39±12.64
	**Z low**				
	**Z high**				
	**C**				
**II/O**	**P**	-10±31.62 -63.33±48.30* -70.00±35.83** -61.67±45.85*	100 ±0.00 -43.33±23.83*** + -31.67±22.84***++ -73.33±28.54***	-12.98±19.81 8.91±6.36** 12.28±9.88*** 19.21±16.49***	-3.80±28.72 -5.59±37.03 -16.77±12.85 -29.46±18.19*
	**Z low**				
	**Z high**				
	**C**				
**II/I**	**P**	20±63.25 -10.00±31.62 -5.00±15.81 -15.00±33.75	0.00±0.00 -10±21.08 -30±34.96 -25±42.49	-6.47±17.39 1.01±3.17 3.72±11.7 3.56±4.91	-6.65±20.18 -1.33±11.59++ -13.15±9.61 -23.95±20.34
	**Z low**				
	**Z high**				
	**C**				

The results are presented as mean ± SD. Statistical comparisons were performed using independent T-test.

P: Placebo group; Z low group were treated with 5 mg/kg/day of the extract; Z high group were treated with 10 mg/kg/day of the extract; C group were treated with 1.2 mg/kg/day carvacrol.

* p<0.05,

** p<0.01, and

*** p<0.001, show significant differences as compared to group P.

+ p<0.05, and

++ p<0.01, show significant differences as compared to group C

## Discussion

The results of two-month treatment of asthmatic patients with two doses of *Z. multiflora* (5 and 10 mg/kg/day) and one dose of carvacrol (1.2 mg/kg/day) revealed clinical eﬃcacy of *Z. multiﬂora* extract and carvacrol on asthma by decreasing lung wheezing, increasing FEV_1_ value and reducing an oxidative marker (NO^2-^). 

Treatment with both doses of *Z. multiflora* and carvacrol for one and two months was effective in improvement of wheezing in asthmatic patients. FEV_1_ values were also significantly increased in patients treated with *Z. multiflora* and carvacrol during this period. In a clinical evaluation, Hosseini et al., applied *Z. multiﬂora* extract syrup and diphenhydramine for the treatment of common cold-induced cough in children and concluded that *Z. multiﬂora* is more useful than diphenhydramine for reducing coughs (Hosseini et al. 2016 ▶). In addition, previous studies suggested a bronchodilatory effect for carvacrol as it demonstrated a relaxant effect on guinea pig tracheal smooth muscles (Boskabady and Jandaghi 2003a ▶; Silva et al., 2014 ▶). The inhibitory effect of the plant and its extract on muscarinic (Boskabady et al. 2011 ▶) and histamine H1 receptors (Boskabady et al. 2012b ▶) as well as their stimulatory effect on β2-adrenoreceptors (Boskabady et al. 2010 ▶) were shown as possible mechanisms of the relaxant effect of the extract and carvacrol on tracheal smooth muscles. All described studies support the findings of the present study indicating the therapeutic potential of *Z. multiﬂora* and its constituent, carvacrol on asthma. 

The results of the current study also showed that *Z. multiﬂora* and carvacrol treatment reduced the plasma levels of NO^2-^ after one and two months of treatment while treatment with placebo did not cause significant changes in NO^2-^ levels. Reactive nitrogen species have cellular deleterious effects and can cause both apoptosis and necrosis and subsequently damage the airways (Ricciardolo et al., 2006 ▶). The immunopharmacological properties of *Z. multiﬂora* have been shown by several *in vitro* and *in vivo* models. In cultured human monocytes, the inhibitory effect of *Z. multiflora* essential oil on nitric oxide (NO) production was exhibited (Kavoosi et al. 2012 ▶). In addition, treatment with carvacrol significantly down-regulated the genes expressions of *TNF-α*, *IL-6*, *iNOS*, and *COX-2*, reflecting the anti-oxidant and anti-inflammatory activity of carvacrol (Aristatile et al. 2013 ▶). Therefore, *Z. multiflora* and carvacrol by decreasing NO in asthmatic patients may improve lung inflammation, the main pathophysiologic characteristic of the disease; through this reduction, *Z. multiflora* and carvacrol can ameliorate disease severity and symptoms.

The percentage of changes in EW, FEV_1_ and NO^2-^ in asthmatic patients following treatment with carvacrol for one and specially two months, were more marked compared to the treatment with both doses of *Z. multiflora*. These results suggest that the therapeutic effect of the plant is perhaps due to the effect of its constituent, carvacrol. 

*Z. multiflora *treatment also led to reduction of WBC, eosinophil, neutrophil and monocyte in blood samples of COPD animals (Boskabady and Gholami Mhtaj 2014 ▶). *Z. multiflora* and carvacrol also increased gene expression of anti-inflammatory (*IFN-γ*, and *FOXP3*) and decreased inflammatory cytokines (*IL-4*, *TGF-β*, and *IL-17*) in sensitized mice (Kianmehr et al., 2016 ▶; Kianmehr et al., 2017 ▶). These studies together with the results of the present study, showed that *Z. multiflora* and carvacrol are effective in the treatment of asthma by reducing lung inflammation by various mechanisms. 

The results of the present study showed that treatment of asthmatic patients with *Z. multiflora* and carvacrol resulted in improvement of respiratory symptoms and PFT values perhaps by reducing lung inflammation. However, to better understand the effect of *Z. multiflora* and carvacrol in asthmatic patients, more clinical trials focusing on evaluation of various inflammatory mediators and cytokines, treatment with longer time period in a larger population, are needed to confirm the therapeutic potential of the plant and its constituent on asthma. Also, we suggest that lower and higher doses of *Z. multiflora* and carvacrol should be examined in further studies. The relatively big SD in our results is due to relative small sample size. However, with the present sample size the significant effect of treatment with the plant and carvacrol on most of the parameters were seen but in the future the effect of *Z. multiflora* and carvacrol should be examined in a larger population of asthmatic patients. 

In conclusion, the results showed improvement in lung wheezing and FEV_1_ value in asthmatic patients who were treated with *Z. multiflora* and carvacrol for two months, which could be due to anti-inflammatory properties of these agents through reduction of NO^2-^. Therefore, a preventive therapeutic effect for the plant on asthma could be suggested which is perhaps due to the presence of its constituent, carvacrol.
